# Advancing Remote Monitoring for Patients With Alzheimer Disease and Related Dementias: Systematic Review

**DOI:** 10.2196/69175

**Published:** 2025-05-14

**Authors:** Mohmmad Arif Shaik, Fahim Islam Anik, Md. Mehedi Hasan, Sumit Chakravarty, Mary Dioise Ramos, Mohammad Ashiqur Rahman, Sheikh Iqbal Ahamed, Nazmus Sakib

**Affiliations:** 1 Department of Electrical and Computer Engineering Kennesaw State University Kennesaw, GA United States; 2 Department of Mechanical Engineering Khulna University of Engineering & Technology Khulna Bangladesh; 3 Louisiana State University Health Sciences Center (LSUHSC) New Orleans School of Nursing Louisiana State University New Orleans United States; 4 Department of Electrical and Computer Engineering Florida International University Miami, FL United States; 5 Department of Computer Science Marquette University Milwaukee, WI United States

**Keywords:** dementia, Alzheimer disease, remote monitoring, Alzheimer, caregiver, fall detection, artificial intelligence

## Abstract

**Background:**

Using remote monitoring technology in the context of Alzheimer disease (AD) care presents exciting new opportunities to lessen caregiver stress and improve patient care quality. The application of wearables, environmental sensors, and smart home systems designed specifically for patients with AD represents a promising interdisciplinary approach that integrates advanced technology with health care to enhance patient safety, monitor health parameters in real time, and provide comprehensive support to caregivers.

**Objective:**

The objectives of this study included evaluating the effectiveness of various remote sensing technologies in enhancing patient outcomes and identifying strategies to alleviate the burden on health care professionals and caregivers. Critical elements such as regulatory compliance, user-centered design, privacy and security considerations, and the overall efficacy of relevant technologies were comprehensively examined. Ultimately, this study aimed to propose a comprehensive remote monitoring framework tailored to the needs of patients with AD and related dementias.

**Methods:**

Guided by the PRISMA (Preferred Reporting Items for Systematic Reviews and Meta-Analyses) framework, we conducted a systematic review on remote monitoring for patients with AD and related dementias. Our search spanned 4 major electronic databases—Google Scholar, PubMed, IEEE Xplore, and DBLP on February 20, 2024, with an updated search on May 18, 2024.

**Results:**

A total of 31 publications met the inclusion criteria, highlighting 4 key research areas: existing remote monitoring technologies, balancing practicality and empathy, security and privacy in monitoring, and technology design for AD care. The studies revealed a strong focus on various remote monitoring methods for capturing behavioral, physiological, and environmental data yet showed a gap in evaluating these methods for patient and caregiver needs, privacy, and usability. The findings also indicated that many studies lacked robust reference standards and did not consistently apply critical appraisal criteria, underlining the need for comprehensive frameworks that better integrate these essential considerations.

**Conclusions:**

This comprehensive literature review of remote monitoring technologies for patients with AD provides an understanding of remote monitoring technologies, trends, and gaps in the current research and the significance of novel strategies for remote monitoring to enhance patient outcomes and reduce the burden among health professionals and caregivers. The proposed remote monitoring framework aims to inspire the development of new interdisciplinary research models that advance care for patients with AD.

## Introduction

### Background

Alzheimer disease (AD) is the most generic form of dementia, and significantly affects the brain cells, declines cognitive abilities, and makes it difficult to perform daily tasks [1]. The main effects of the disease are loss of memory, inability to use problem-solving and logical thinking skills, anxiety, depression, and confusion [2]. AD has remained the leading cause of death among people aged >60 years. The main cause of AD is the accumulation of abnormal proteins such as β-amyloid and microtubule-associated tau protein in the brain; the aggregation of these proteins causes synaptic disintegration and neural loss, which characterize AD [3,4]. Disturbingly, it is estimated that the number of AD cases could grow to 13.8 million by 2060. The pervasive nature of this disease underscores the pressing need for continued research, intervention strategies, and support systems to address the growing challenges posed by AD in an aging population [5,6].

Caring for individuals with AD is an enduring and demanding journey, often placing a significant burden on family members who become primary caregivers or on those who opt to hire professional caregivers [7]. The responsibilities encompass a spectrum of care, requiring a deep commitment to providing the essential support needed for patients with AD to navigate their daily lives. Caregivers play a crucial role in assisting individuals with AD in tasks integral to their routine while also implementing lifestyle adjustments aimed at alleviating stress, confusion, anxiety, and agitation. The caregiving journey involves fostering an environment that promotes mental, physical, and social well-being. This includes implementing tailored strategies to keep patients with AD mentally engaged, encouraging physical activities that align with their abilities, and facilitating social interactions to prevent isolation. However, continuous monitoring and provision of care for patients with AD causes a substantial burden on both the family members and caregivers [8,9]. This responsibility often causes a lot of stress and depression among those providing care. Family members and caregivers find themselves dealing with the progressive loss of individuals with AD. In addition to that, they must navigate between caregiver responsibilities and sustaining a work-life balance. Disputes between family members, financial burdens, and escalating medical expenses extend the challenges that those undertaking caregiver roles face [10]. While existing research has identified various scales to measure the burden on family members, there is a notable gap in the literature concerning effective strategies to alleviate this burden. Manzini and do Vale [11] and Liao et al [12] address this critical gap by exploring remote monitoring methods facilitated by ITs. By leveraging innovative approaches, the research seeks to enhance the quality of care provided to patients with AD and substantially reduce the burden experienced by family members and caregivers. Through the implementation of such technological solutions, the aforementioned studies [11,12] aspired to contribute valuable insights into improving the overall well-being of both patients with AD and those entrusted with their care.

Despite the growing body of research on remote monitoring for AD and related dementias (ADRD), significant gaps remain in the literature, particularly regarding interdisciplinary integration. Previous reviews have primarily focused on individual aspects of remote monitoring, such as wearable devices [13-16], smart home sensors [17-19], or privacy and security considerations [20,21], often in isolation. However, the effectiveness of these technologies depends on a holistic approach that integrates expertise from health care, computer science, cybersecurity, and user-centered design. The lack of interdisciplinary research has led to fragmented solutions that may not fully address the practical needs of patients and caregivers. This review sought to bridge these gaps by offering a comprehensive assessment of remote monitoring technologies while emphasizing their usability, security, and ethical implications. Therefore, we formulated four research questions (RQs) to explore existing remote monitoring technologies and their applications, assess their needs and advantages, examine privacy concerns related to remote monitoring, and investigate technology adoption among older adults:

- RQ 1: What are the primary remote monitoring technologies to capture different behavioral, physiological, and environmental data?
- RQ 2: To what extent do these existing remote monitoring technologies achieve an optimal balance of efficiency, practicality, and empathy in addressing the needs of both patients and caregivers?
- RQ 3: What are the critical security and privacy considerations and concerns involved in these remote monitoring technologies?
- RQ 4: What are the critical design considerations and technology components for a comprehensive remote monitoring framework specifically tailored to patients with AD?

While this review focused on remote monitoring for individuals with AD, it is important to acknowledge that many of the challenges, technologies, and solutions discussed are also relevant to other dementia subtypes. Conditions such as vascular dementia [22,23], Lewy body dementia [24], and frontotemporal dementia [25] share overlapping symptoms [26,27], including cognitive decline, behavior changes, and mobility issues, which can similarly benefit from remote monitoring interventions. However, our decision to center this review on AD was guided by its high prevalence and the significant body of research available on remote monitoring solutions tailored specifically to ADRD. The insights presented in this paper may be broadly applicable to dementia care, although future research should further explore technology adaptations for different dementia subtypes.

The remainder of this paper is divided into 3 key sections. Section 2 outlines the methodology, comprising 3 subsections that detail the scoping criteria, literature search strategy, and data analysis procedures. Section 3 addresses the RQs in depth. Finally, section 4 concludes the literature review, summarizing the key findings and their implications.

### Objectives

Our objective with this paper was to critically assess the effectiveness of various remote sensing technologies in enhancing patient outcomes, particularly for individuals with ADRD, while also identifying strategies to reduce the burden on health care professionals (HCPs) and caregivers. To perform this assessment, the research comprehensively examined key factors essential for the successful implementation and adoption of these technologies. These factors include patient and caregiver compliance, ensuring that the technologies are used consistently and effectively; user-centered design, which focuses on creating accessible and intuitive systems tailored to the specific needs of end users; privacy and security concerns, addressing the safeguarding of sensitive patient data and ensuring adherence to ethical and regulatory standards; and the overall efficacy of these technologies, evaluating their accuracy, reliability, and impact on patient care.

## Methods

### Overview

In the landscape of AD care, this review undertook a comprehensive examination of the complex dynamics introduced by remote monitoring technologies. We focused on different remote monitoring technologies, types of algorithms used to process the data, issues and concerns related to security and privacy, caregiver burdens, and specific monitoring frameworks for patients with AD. We conducted an extensive search to identify relevant research papers across multiple disciplines from 2011 to 2024. Our search encompassed a diverse array of digital libraries, including Google Scholar, IEEE Xplore, PubMed, and DBLP on February 20, 2024, with an updated search on May 18, 2024, to ensure coverage across multidisciplinary domains, including health care, engineering, and computer science. These databases were selected based on their relevance to remote monitoring technologies, security and privacy considerations, and AD care. While Scopus, Embase, and Cochrane are widely recognized for systematic reviews in clinical and biomedical research, our focus on technological, interdisciplinary, and engineering aspects made IEEE Xplore and DBLP particularly valuable sources. In addition, PubMed was included to ensure coverage of relevant medical and health care studies. Google Scholar was used to capture a broader range of studies, including conference proceedings and gray literature, which are crucial for emerging technologies. On Google Scholar, the search string used was *“Remote monitoring technologies” AND “Wearable sensors” AND Alzheimer AND “Privacy concerns,”* ensuring a broad examination of related literature. PubMed and IEEE Xplore were queried using the search string *(Remote monitoring technologies) AND Alzheimer AND (“Privacy concerns”)* to capture articles with a focus on technical and medical perspectives. In addition, DBLP was queried using the search string *Alzheimer AND “Remote monitoring”* to include publications emphasizing computer science and engineering insights. In addition, we cross-referenced the citations of each article to uncover additional relevant articles or government reports aligning with our inclusion criteria. Notably, we imposed some constraints on the period during which the papers were published, and only English-language papers were considered. In total, we identified 31 articles that fulfilled our inclusion criteria.

Our search strategy included keywords such as “Remote monitoring technologies” AND “Wearable sensors” AND “Alzheimer” AND “Privacy concerns” to ensure the inclusion of studies addressing security and data protection—critical challenges in adopting remote monitoring solutions. However, we acknowledge that this keyword choice may have limited the retrieval of studies focusing on broader aspects, such as clinical effectiveness, usability, and caregiver perspectives. While our selection process incorporated additional screening to identify studies covering a wider range of topics, future research could benefit from refining the search terms to ensure a more balanced representation of different remote monitoring challenges.

### Study Identification and Selection

A total of 79 papers were initially identified based on their titles and abstracts that were deemed to be potentially relevant to the research topic. Of the 61 records assessed for eligibility, 30 (49%) were excluded for various reasons: 7 (23%) were books, 6 (20%) were partial articles, and 17 (57%) were out-of-context studies. Finally, 31 papers were identified to extract information and insights related to the RQs. These papers were thoroughly analyzed to identify common themes, methodologies, findings, and gaps in the existing literature. This analysis provided a deeper understanding of the current research and valuable insights to guide this review. This systematic approach to literature review and analysis enhanced the validity and reliability of the research, contributing to its overall credibility.

In the following sections, the findings from the papers are presented and discussed. The results are organized based on the information identified in the literature review with respect to the RQs.

## Results and Discussion

### Primary Remote Monitoring Technologies (RQ 1)

#### Overview

**Figure 1:**
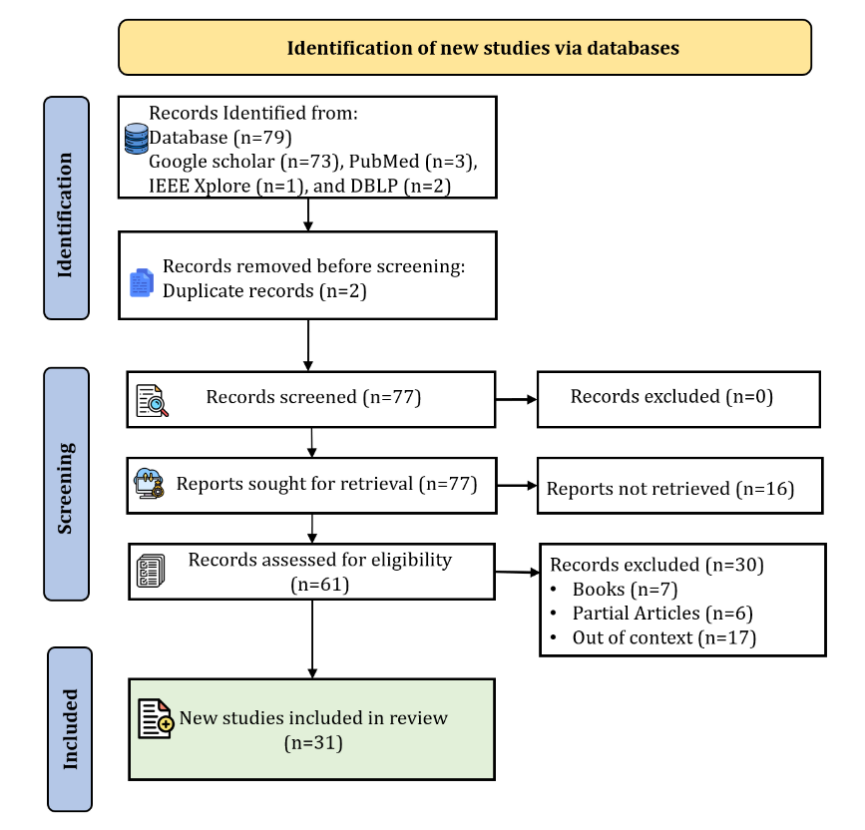
PRISMA (Preferred Reporting Items for Systematic Reviews and Meta-Analyses) flow diagram for the study selection in the literature review.

**Figure 2:**
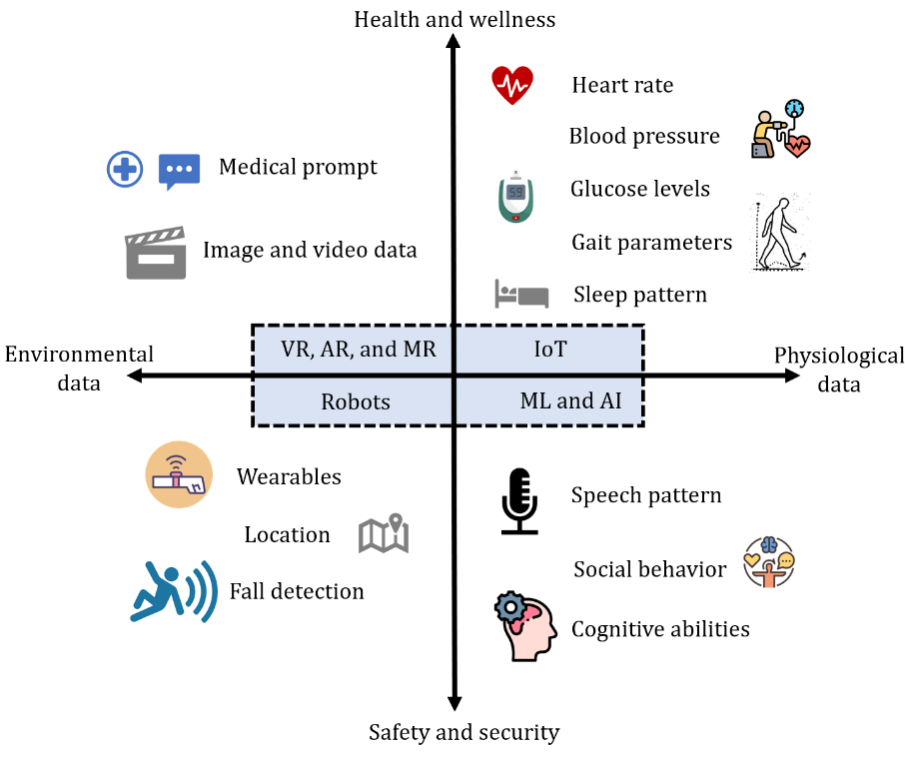
Integration and synergy of different remote monitoring technologies. AI: artificial intelligence; AR: augmented reality; IoT: Internet of Things; ML: machine learning; MR: mixed reality; VR: virtual reality.

Remote monitoring technologies using wearable and portable device–based sensors have revolutionized health care by enabling continuous and real-time tracking of various physiological and environmental parameters. These sensors, embedded in devices such as smartwatches, fitness bands, and portable health monitors, can measure heart rate, blood pressure, glucose levels, temperature, and physical activity [28,29]. They offer significant advantages in managing chronic conditions, detecting early signs of health deterioration, and providing personalized care. Wearable sensors are particularly beneficial in monitoring older patients and individuals with chronic diseases, allowing for prompt interventions and reducing hospital visits. Furthermore, the integration of these devices with mobile health (mHealth) apps and cloud-based platforms facilitates seamless data collection, analysis, and sharing with health care providers, enhancing patient engagement and improving health outcomes. The subsequent subsections provide a detailed description of the various aspects of remote monitoring technologies. To ensure a systematic approach, a thorough review of the literature was conducted following the PRISMA (Preferred Reporting Items for Systematic Reviews and Meta-Analyses) framework [30,31] (Multimedia Appendix 1), as shown in Figure 1. Figure 2 illustrates the integration and synergy of different remote monitoring technologies based on relevant parameters. These are further elaborated on in the following subsections and in Multimedia Appendices 2-5 [14,32-52].

#### Wearables

Wearable technologies refer to devices worn on the body either as an accessory or as clothing. Devices embedded with an accelerometer and gyroscope, such as wrist or ankle devices, are used for analyzing gait parameters such as speed, variability, and stride length [32]. There are also wearable devices that capture physical activity data, including steps taken, intensity, and duration, aiding in tracking physical activity, which helps the patient with AD be physically active and slow down the progression of the disease [32]. In addition, wrist devices and smart clothes are equipped with sensors, whereas various wearables, including wristbands, smartwatches, and chest straps, collect different data such as physiological signs, posture, gait, movement, and sleep data, and enable the continuous monitoring of blood pressure. Devices such as the Basis Health Tracker, Misfit Shine, Fitbit Flex, Withings Pulse O2, and Actiwatch Spectrum actigraph are used for sleep detection and monitoring daily activities [53-57]. These devices enable continuous monitoring of vulnerable patients, offering medical specialists a comprehensive view of the patients’ health for more accurate diagnosis and treatment [33,34]. Moreover, dream headbands are used to record and store physiological data in real time during sleep, facilitating the monitoring of daily sleep patterns and enabling patients and caregivers to access their reports freely [35]. Further details are provided in Multimedia Appendix 2 [32-35,37-45] and Multimedia Appendix 3 [32-34,36-38,42,45-47].

#### Environment and Home Sensors

Remote monitoring through advanced environment and sensor technology provides a promising approach to enhancing care, monitoring daily activities, and detecting early signs of cognitive decline. In the context of home-based remote monitoring, various sensors are used to capture and analyze vital health and activity data. Infrared sensors strategically placed throughout the home facilitate gait analysis by detecting individual movements and providing insights into gait characteristics such as walking speed, rhythm, and variability [32]. Wireless mattress sensors and ambient sensors are used to monitor sleep time, movements, and patterns, aiding in the early detection of mild cognitive impairment (MCI) and AD. In addition, night monitoring systems equipped with motion detectors are used to monitor sleep patterns and alert caregivers to unusual nighttime behavior [32]. In the case of continuous physical activity monitoring, infrared sensors are used to collect internal movement data, which allows for the assessment of activity level without the need for active participation of the individual [32,36]. A multisensory approach, including sound sensors, video cameras, radio-frequency identification readers, pressure sensors, light sensors, proximity sensors, temperature sensors, humidity sensors, and location trackers, has been applied to many activities and environmental conditions in the home [33]. This rich dataset facilitates the assessment of disease progression and provides valuable information about changes in daily activities and environmental factors. Behavior patterns are tracked using sensors installed in various areas of the home, including the kitchen, bedroom, and bathroom, along with fall detection sensors. These sensors help monitor mobility, stability, frequency, and time spent in different areas, contributing to a deeper understanding of daily routines and potential risks [32]. Microsoft Kinect cameras are specifically used for gait monitoring to detect and assess falls among older individuals, with the collected video data aiding in fall prevention efforts [37].

Finally, motion sensor–activated light paths and bed pressure sensors are used for fall prevention and sleep problem detection, respectively. These sensors provide an additional layer of safety and monitoring, enhancing the overall effectiveness of the remote monitoring system [34].

#### Personal Devices

Personal devices, specifically, smartphones equipped with advanced sensors such as accelerometers, gyroscopes, and magnetometers, play a pivotal role in gait analysis and physical activity monitoring. Apps installed on these smartphones analyze the sensor data to determine various gait characteristics, including stride length, walking speed, and rhythm, providing valuable insights into mobility patterns [32]. Moreover, waist-worn accelerometers facilitate continuous monitoring of physical activity parameters such as movements and acceleration, aiding individuals in maintaining optimal levels of physical activity and energy use [32]. In addition to physical activity monitoring, smartphones serve as effective tools for assessing social behavior. By monitoring calls, SMS text messages, and internet-browsing activities, these devices offer insights into an individual’s social activity levels and patterns. This information is crucial for health care providers and caregivers to understand the social interactions of individuals, identify potential signs of isolation, and implement interventions to promote social engagement and well-being [37].

#### Machine Learning and Artificial Intelligence

Machine learning and artificial intelligence (AI) play a pivotal role in health care by leveraging diverse data sources to enhance diagnostic capabilities, predict disease progression, and support clinical decision-making. Driving patterns collected through GPSs are analyzed to detect early signs of preclinical AD. These patterns are compared with disease progression to identify behavior changes linked to AD [32]. Activity data from sensor-embedded devices such as wearables are used for data mining and predictive modeling, aiding in identifying potential health issues and informing health care decisions [46]. Sleep patterns collected from these devices offer valuable insights into an individual’s health status, providing clinicians with early warnings of health deteriorations or relapses and enabling timely interventions to improve patient outcomes [47].

Speech pattern analysis using data from health records, including calls and SMS text messages, offers diagnostic capabilities for neurodegenerative diseases such as Parkinson disease and AD, enabling early detection and intervention [38]. In addition, AI-powered image and video processing techniques assist in diagnosing various conditions, such as cancer from x-ray and computed tomography scans and degenerative diseases from fundus photography, enhancing diagnostic accuracy and treatment planning [38]. Furthermore, data collected from wrist-worn actigraphy devices such as the ActiGraph wGT3X are analyzed using a variety of machine learning algorithms, including logistic regression, random forest, gradient-boosting machine, and support vector machine. These algorithms help predict the risks and progression of disease, providing valuable insights for personalized health care management and intervention strategies [45].

#### Robots

Robots such as Nao, Pepper, Paro, Stevie, Zoro, Mini, and Tangy are emerging as valuable tools in health care settings, particularly for supporting the needs of older adults. These robotic companions engage older individuals through interactive activities such as conversations, quizzes, tongue twisters, and arithmetic calculations, demonstrating promising results in enhancing social engagement and alleviating feelings of isolation [46]. AI-powered robots equipped with advanced sensors and algorithms can access environmental data and user-specific characteristics to deliver personalized medical prompts and emotional support. These robots play a crucial role in physical rehabilitation, reducing feelings of isolation and promoting overall well-being by providing companionship and interactive engagement [38,42]. Furthermore, specialized robots such as Nao, Pepper, and Paro are designed to cater to the unique needs of patients with dementia with cognitive impairment. These robots offer a comprehensive range of services, including behavioral monitoring, physical exercise tutoring, recreational activities, stress management, and medication reminders. By providing multifaceted support, these robotic companions significantly enhance the quality of life of older adults and contribute to improved health care outcomes [34]. Robots named Stevie and Zoro are specially tailored to support caregivers and older adults with memory loss. These robots offer care support, entertainment options, cognitive engagement activities, and social connectivity features. Their interactive nature stimulates older adults through engaging exercise and interaction, fostering a sense of community and connection [32]. Robots such as Mini and Tangy enhance cognitive abilities in older adults using educational games and imitation learning, aiming to improve memory, cognitive function, and mental agility through innovative stimulation and support [37].

#### Internet of Things

The Internet of Things (IoT) has revolutionized health care monitoring by using novel sensors and wireless communication methods such as Bluetooth, Wi-Fi, near-field communication, and Zigbee to access and securely store real-time data on vital signs, including temperature, blood pressure, heart rate, and glucose levels, in the cloud. This advancement facilitates effective device management and real-time data monitoring and offers a secure storage solution, enhancing patient care and treatment outcomes [47]. Furthermore, the integration of multiple sensors into wearable devices, environment monitoring systems, and in-home setups provides pervasive and unobtrusive intelligence to support individuals proactively in their daily lives. These sensors enable continuous monitoring of various parameters, including temperature, humidity, and blood pressure, offering valuable insights for personalized health care interventions [39,42].

IoT-based tools such as smart pillboxes assist individuals in medication management by sending reminders according to prescribed medication plans, ensuring adherence to treatment regimens, and improving medication compliance [39]. Moreover, the implementation of semantic frameworks and advanced data processing techniques enables the collection and analysis of multi-sensor data to determine disease progression and provide timely alerts through real-time monitoring. This approach aids in the early detection of health deterioration, facilitating timely interventions and improved patient outcomes [40]. In addition, the DCARE model used a computational approach to process data from monitoring devices, incorporating content prediction, behavior recognition, and content recognition. This model serves as a comprehensive solution for general monitoring, data processing, and sending alerts to caregivers in case of potential risks or emergencies, enhancing patient safety and care coordination [41].

#### Virtual, Augmented, and Mixed Reality

Virtual reality, augmented reality, and mixed reality technologies, facilitated by devices such as headsets, smart glasses, and next-generation smartphones, offer an immersive experience that extends beyond entertainment to include web-based home shopping, leisure activities, and communication, enriching both social interaction and physiological well-being [42]. Smart spectacles equipped with machine learning algorithms for face and object recognition enable personalized video and image playback, assisting patients with dementia and memory loss by stimulating memory recall and cognitive engagement [43]. In addition, GPS and device tracking functionalities, combined with medication reminders via Bluetooth connectivity, empower caregivers to monitor patient locations and medication adherence, ensuring timely interventions and enhancing patient safety [44].

### Effectiveness of Remote Monitoring Technologies (RQ 2)

#### Overview

**Figure 3:**
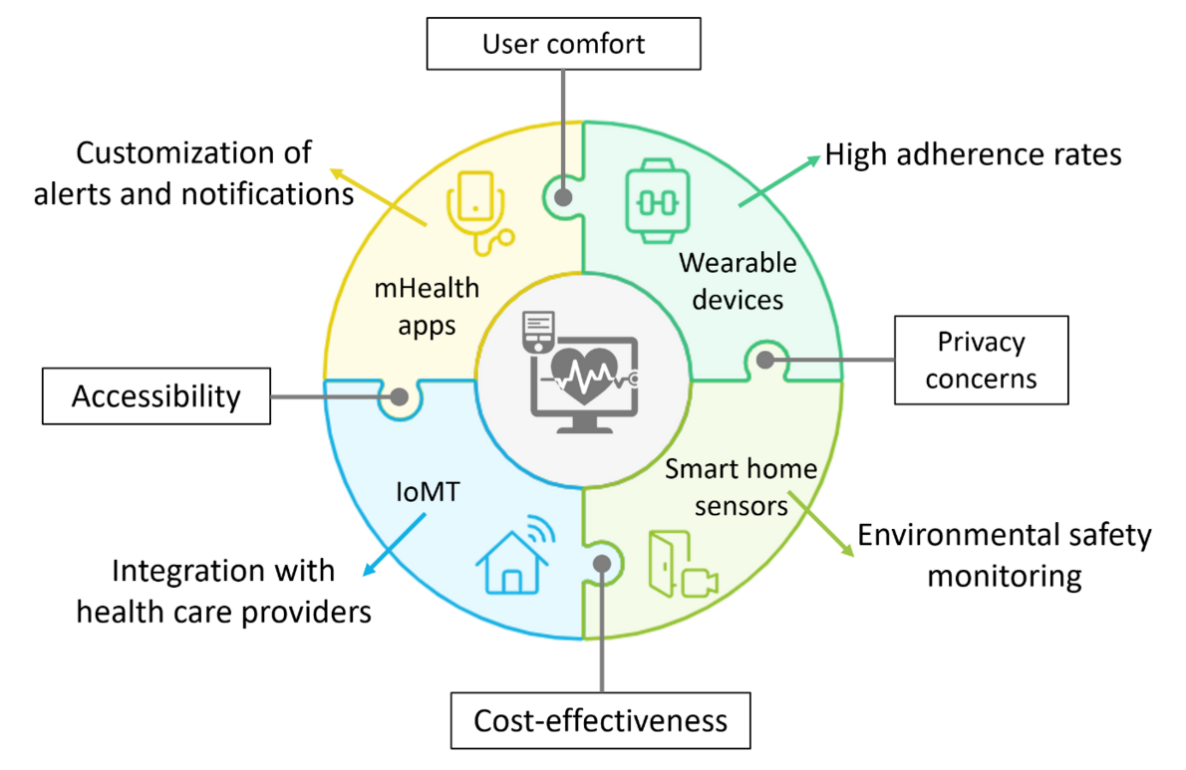
Key features and considerations of different remote monitoring technologies. IoMT: Internet of Medical Things; mHealth: mobile health.

In addressing RQ 2, which investigated the effectiveness of remote monitoring technologies for individuals with ADRD, a thorough review of previous research papers was conducted. The findings are synthesized and summarized in Multimedia Appendix 4 [14,32,33,36-38,42,45,46,48-52] and Figure 3, providing a detailed comparison of various monitoring methods, their advantages, and their limitations. This comprehensive assessment helps achieve an optimal balance in addressing the needs of both patients and caregivers. The following subsections delineate different aspects pertaining to RQ 2.

#### Wearable Devices

Wearable devices have emerged as valuable tools for remote monitoring of various health conditions, including cerebrovascular diseases, neurodegenerative diseases, and MCI. Godkin et al [48] investigated the adherence value of wearable devices focusing on 3 key aspects—adherence based on sensor location, time of day, and wear location—and participant and study partner acceptance of continuous multi-sensor wear for a week-long period. The outcomes of this study demonstrated the feasibility of a continuous multi-sensor approach to remote monitoring for individuals living with cerebrovascular diseases and neurodegenerative diseases, showing high adherence rates during both day- and nighttime wear throughout the study period. The aforementioned study also addressed concerns related to device discomfort, including itchiness, catching on clothing during dressing or on bed sheets during sleep, device size, and appearance.

Similarly, a comprehensive investigation conducted by Stavropoulos et al [14] focused on public involvement activities to capture the preferences, priorities, and concerns of people with AD and their caregivers regarding the use of monitoring wearables. Face-to-face discussions with the patient advisory board revealed varying levels of acceptance toward new technologies among older individuals, highlighting concerns regarding the cost of devices, adaption to modern technologies, and potential difficulties in operating technology. Furthermore, Stavropoulos et al [14] used a questionnaire to understand the specific requirements, preferences, barriers, and daily obstacles faced by individuals with MCI, their caregivers, and HCPs in using wearables. The Human Factors and Technology Requirements Questionnaire collected survey responses to various types of questions, such as open-ended, multiple-choice, and closed-ended questions, and the importance of user-centered design for assistive technologies using wearables and highlighted the implications for future research. The aforementioned study provided valuable insights into the perspective of individuals with MCI, caregivers, and HCPs regarding the use of assistive technologies with wearable trackers, emphasizing the need to consider privacy and ethical issues in the technology design and the disadvantages of traditional record-keeping methods.

#### Smart Homes (Multiple Sensors)

Smart home technologies equipped with multiple sensors have shown promising benefits in remote monitoring, particularly through video monitoring methods. However, there is a growing interest in exploring novel remote monitoring technologies beyond traditional video monitoring, emphasizing the need to diversify research in this area [36]. Despite the potential advantages, there is limited evidence to conclusively determine the acceptance of these remote sensing technologies, indicating a gap in understanding alternative methods [36]. In a comprehensive systematic review, it was highlighted that smart home remote monitoring has the potential to support older persons and caregivers by addressing individual family needs [49]. Nevertheless, critical barriers need to be addressed for successful adoption and implementation, including concerns about the time, training, and technical challenges associated with smart home technologies [49]. These challenges include issues with battery life, unstable wireless connectivity, and incompatibility, which have raised concerns about potential feelings of loneliness and isolation among older people with cognitive impairments [49]. Furthermore, there is a need for more research on alternative remote sensing technologies, such as vibration and acoustics technologies, to better understand their potential applications and effectiveness [36].

Addressing stigma and negative associations with disability through appropriate design and customization of sensing technologies, such as the use of smart fabric sensors, could help mitigate privacy concerns and enhance acceptability [36]. Future research should focus on understanding end-user conditions for acceptability and addressing various barriers, including usability, technical issues, cost concerns, and social implications, to facilitate the broader adoption of smart home technologies in health care settings [36,49].

#### Multimode Sensor (Machine Learning Approach)

In addressing the challenges of predicting and managing the behavioral and psychological symptoms of dementia (BPSD), multimode sensor technologies coupled with machine learning approaches have emerged as promising tools [45]. These approaches leverage diverse data sources to offer valuable insights into the relative contributions of various factors, such as caregiver-perceived triggers, cognitive and functional status, personality traits, and actigraphy-derived parameters, in predicting BPSD. However, the efficacy of these technologies can be compromised by challenges related to the improper wear of devices, leading to limited data availability for accurate prediction [45]. Despite these obstacles, machine learning models have demonstrated their capability to effectively predict different subsyndromes of BPSD. Such predictive capabilities hold significant potential to inform personalized interventions tailored to individual symptom profiles, thus facilitating timely and targeted care.

Nevertheless, it is essential to acknowledge the existing limitations and gaps in current research [45]. Cho et al [45] emphasize the need for future studies to address these challenges. This includes validating findings in larger and more diverse populations, integrating objective monitoring technologies, and developing more precise prediction algorithms to enhance the accuracy and reliability of these predictive models. Such advancements could significantly contribute to improving the quality of care and support provided to individuals living with dementia and their caregivers.

#### Patient and Public Involvement Survey

Patient and public involvement surveys emphasize the importance of prioritizing user-friendly design and personalized approaches when developing mHealth apps tailored for individuals with cognitive impairment [50]. Survey findings in the study by Lazarou et al [50] highlighted participants’ keen interest in brain games aimed at memory enhancement, showcasing a strong demand for cognitive support tools. In addition, the participants expressed a desire for medication reminders and GPS tracking functionalities within mHealth apps, demonstrating the potential utility of such features in enhancing daily living and emergency response for individuals with cognitive impairment [50]. Despite these valuable insights, it is important to acknowledge the survey’s limitations. The research was conducted among a limited population, potentially introducing self-bias and not capturing the full spectrum of needs and preferences across diverse groups [50]. To address these challenges, future research should aim to explore more diverse populations and incorporate a broader range of user perspectives. This will help in developing more inclusive and effective mHealth interventions that cater to the diverse needs of individuals with cognitive impairment, caregivers, and HCPs.

#### Internet of Medical Things and Robots

The Internet of Medical Things (IoMT) is revolutionizing the health care landscape by integrating advanced technologies into smart health care systems. In the study by Mathkor et al [52], the authors provided a comprehensive overview of IoMT, highlighting its role in current and future biomedical systems. The study emphasized the integration of technologies such as deep learning, machine learning, blockchain, AI, radio-frequency identification, and Industry 5.0. These technologies hold immense potential in enhancing patient care, improving diagnostic accuracy, and streamlining health care operations. However, the implementation of IoMT systems presents several challenges that need to be addressed for successful adoption. High costs associated with device development, infrastructure setup, and connectivity solutions can pose financial barriers. In addition, many IoMT devices operate on battery power, leading to concerns about battery life and continuous operation. Data quality issues, including noise, biases, and inaccuracies, can also arise due to device limitations or environmental factors. To fully realize the benefits of IoMT, it is crucial to address these challenges and develop robust, scalable, and secure solutions. Companion robots have emerged as potential tools to address the social and emotional needs of older adults, particularly those with dementia.

In the study by Pou-Prom et al [51], the authors conducted an experiment involving 19 participants diagnosed with AD to explore the feasibility of using conversational companion robots. The study revealed that, while some participants enjoyed interacting with the robots, others expressed doubtfulness and reluctance. Technical limitations such as speech recognition challenges, lack of dialogue context, and keyword matching were identified as barriers to seamless communication. Furthermore, the absence of features such as miscommunication and acoustic features hindered the assessment of dementia-related symptoms through robot interactions. The mixed reactions from participants highlighted the importance of ongoing research to refine companion robot technologies. Future work should focus on addressing the identified limitations, exploring the trade-off between autonomous and semiautonomous approaches, and optimizing the design and functionality of these robots to better support the well-being of older adults. Collaborative efforts involving HCPs, engineers, and end users are essential to develop more effective and user-friendly companion robot solutions that can have a meaningful impact on the lives of older adults with dementia.

#### Cognitive Monitoring for Dementia Care

Advancements in remote monitoring have enabled the integration of cognitive assessment tools to track disease progression and evaluate treatment effectiveness in dementia care. Digital cognitive assessments such as smartphone-based memory and attention tests offer frequent, scalable evaluations that provide real-time insights into cognitive changes. In addition, AI-driven speech and typing analysis can detect subtle linguistic and motor impairments associated with cognitive decline, enabling early intervention and treatment adjustments. Gaze tracking and eye movement analysis further enhance cognitive monitoring by assessing reading patterns, recognition abilities, and attention shifts, which may indicate neurological changes over time. Meanwhile, wearable electroencephalography devices and neurofeedback tools provide continuous, objective measurements of brain activity, allowing researchers and clinicians to assess how therapeutic interventions impact cognitive function. These emerging technologies hold great promise for improving personalized treatment strategies, monitoring drug efficacy, and facilitating early diagnosis, ultimately enhancing the quality of care for individuals with dementia.

### Privacy and Security in Remote Monitoring (RQ 3)

#### Overview

When examining the critical security and privacy considerations involved in remote monitoring technologies, particularly for patients with AD, it is essential to delve into several layers of complexity that encompass data security, privacy regulations, ethical issues, and the unique vulnerabilities of the patient population. Remote monitoring technologies, including smartphones, wearables, and environmental sensors, offer significant benefits for managing AD by enabling continuous health monitoring and early detection of anomalies and providing valuable data for research. However, these benefits come with substantial security and privacy challenges. Different aspects of privacy and security concerns are summarized in Multimedia Appendix 5.

#### Data Types and Associated Risks

First, the nature of the data collected by these devices—ranging from personal details to medical records, geolocation, and behavioral patterns—makes them highly sensitive. Ensuring data security is paramount as it requires robust encryption methods to protect data both in transit and at rest. Encryption safeguards the data from unauthorized access during transmission and storage. Equally important is implementing strong access control measures, ensuring that only authorized individuals such as health care providers and approved caregivers can access the patient data. Regular security audits and vulnerability assessments are crucial to identifying and addressing potential security gaps, thereby maintaining the integrity of the monitoring systems.

Privacy concerns are equally critical, particularly given the stringent requirements set forth by regulations such as the General Data Protection Regulation (GDPR) in Europe and HIPPA (Health Insurance Portability and Accountability Act) in the United States [58-60]. These regulations mandate that patients’ personal health information be protected and that individuals have control over their data. Adherence to these regulations involves ensuring that patients (or their legal guardians) provide informed consent for data collection and use. This consent process must be clear and comprehensive, detailing what data will be collected, how they will be used, who will have access, and how long the data will be retained. Data minimization principles should be applied, collecting only the necessary data required for the intended purpose, thus reducing the risk of unnecessary exposure. Patients with AD may not fully understand the implications of data collection and their rights regarding data privacy. Therefore, it is imperative to involve caregivers or legal guardians in the consent process to ensure that the patients’ rights and preferences are respected. Furthermore, patients with AD are particularly vulnerable to exploitation and abuse. Continuous monitoring can inadvertently infringe on their privacy and autonomy, raising ethical concerns. Balancing the need for constant monitoring to ensure safety with the patients’ rights to privacy and dignity is a delicate task. Ethical considerations must guide the implementation of these technologies, ensuring that monitoring practices do not become overly intrusive.

#### Device and IoT Security

From a technical perspective, the integration of various monitoring devices such as smartphones, wearables, and environmental sensors introduces additional security challenges. Each device and sensor can be a potential entry point for cyberattacks. IoT devices are often targeted due to their typically lower security standards. To counteract these risks, it is crucial to ensure that all devices are secured against tampering and hacking. This involves regular firmware updates to patch vulnerabilities, the use of strong authentication mechanisms to prevent unauthorized device access, and the use of data anonymization techniques to protect patient identity in the event of a data breach [41,61].

Furthermore, the use of advanced technologies such as AI and machine learning in processing and analyzing the collected data presents its own set of risks. While these technologies can enhance the accuracy and efficiency of health monitoring, they also require access to large datasets, which can exacerbate privacy concerns if not handled properly. Ensuring that AI and machine learning models are trained on anonymized data and that they adhere to strict privacy standards is essential to mitigate these risks. Potential attackers pose significant threats to the security of remote monitoring systems. These attackers might attempt to intercept data transmission, gain unauthorized access to monitoring devices, or exploit vulnerabilities to extract sensitive information. Addressing these threats requires a multilayered security approach, including end-to-end encryption, secure communication protocols, and robust incident mechanisms to quickly address and mitigate breaches when they occur [47].

#### Balancing Safety and Patient Autonomy

While remote monitoring offers substantial benefits for managing AD, it also presents critical security and privacy challenges that must be carefully addressed. Ensuring robust data security through encryption, access controls, and regular audits; complying with privacy regulations; obtaining informed consent; and addressing the unique vulnerabilities of patients with AD are essential components of a secure and ethical monitoring system [62,63].

Balancing safety and patient autonomy in remote monitoring for AD care presents a complex challenge that requires a nuanced approach. While the technology offers substantial benefits, such as continuous health monitoring, early anomaly detection, and timely interventions, it must be implemented thoughtfully to prevent undue intrusion. The need for constant observation can infringe on the patients’ sense of independence, raising ethical questions about dignity and consent. To address these concerns, remote monitoring systems should integrate customizable privacy settings that allow caregivers to modify the monitoring level based on the patients’ current health status and preferences. Noncritical data anonymization and local data processing using edge computing can significantly reduce the sense of surveillance, ensuring that only essential information is transmitted to central servers [64].

Obtaining informed consent from patients with AD is another critical aspect due to cognitive impairments that often affect their decision-making capabilities. In such cases, involving caregivers or legal guardians becomes essential to uphold the patients’ rights while ensuring that their best interests are protected [65,66]. Technologically secure consent management systems can be used to store and track consent records digitally. Leveraging blockchain technology for tamper-proof consent logs can enhance transparency and accountability, making it clear who has agreed to data collection and how the data will be used. This approach ensures compliance with regulations such as the GDPR [60] and HIPAA [58,67], reinforcing trust among patients, caregivers, and health care providers.

Adaptive monitoring systems that support variable levels of oversight can help maintain patient autonomy by shifting from comprehensive tracking to minimal oversight as needed. This adaptability allows caregivers to balance the safety needs with the patients’ right to privacy. Moreover, implementing user-controlled privacy features empowers patients and their caregivers to configure data sharing preferences, such as choosing which types of data are collected and how frequently they are transmitted. This flexibility can significantly improve user satisfaction and trust in the monitoring system.

AI integration into monitoring systems should prioritize ethical standards, ensuring that data handling is minimal and transparent. For instance, using federated learning allows algorithms to be trained directly on local devices, avoiding the need to transfer raw data to external servers [68]. This method safeguards privacy while maintaining the system’s ability to deliver valuable insights. In addition, explainable AI frameworks can provide justifications for alerts and decisions, ensuring that caregivers understand the reasoning behind the system’s actions [69]. Such transparency bolsters confidence in the technology and facilitates informed decision-making.

To enhance safety protocols without compromising privacy, remote monitoring systems should use multilayered privacy protocols that combine encryption, robust user authentication, and differential privacy [70]. By injecting noise into datasets, differential privacy makes it difficult to trace data back to individual patients while allowing for meaningful analysis. Regular security audits and ethical reviews should also be conducted to ensure that monitoring practices do not become overly invasive. Independent ethics committees can provide oversight, ensuring that the technology aligns with patient-centric values and preserves autonomy.

Maintaining patient dignity is paramount, especially when using advanced monitoring technology. Nonvisual sensors such as microradar [71] systems can detect falls and wandering without capturing images or videos, offering a less invasive alternative to camera-based surveillance. Moreover, patients and caregivers should have the option to customize notification settings, choosing to receive only high-priority alerts to minimize stress. This selective alert system can enhance quality of life by allowing patients to maintain a degree of normalcy and autonomy in their daily lives.

### Potential Remote Monitoring Framework for Patients With AD (RQ 4)

#### Overview

Critical design considerations for a potential remote monitoring framework for patients with AD include ensuring privacy and data security to comply with HIPAA regulations, using nonintrusive monitoring technologies such as microradar sensors to maintain patient dignity, and incorporating AI-driven analytics for accurate detection of falls and wandering. The system should offer real-time alerts to caregivers, be user-friendly for both patients and caregivers, and integrate with existing health care platforms for seamless data sharing. In addition, providing customizable and scalable features can accommodate diverse patient needs and facilitate broader adoption.

The development of our proposed remote monitoring framework was directly informed by the insights gained from our literature review. Our findings revealed that existing monitoring solutions such as wearable sensors and camera-based systems present significant limitations in terms of user compliance, privacy concerns, and real-time responsiveness. Wearable devices require active patient participation, which may not always be feasible for individuals with ADRD, whereas camera-based systems raise ethical and privacy concerns. In response, our framework integrates microradar technology, which allows for the nonintrusive monitoring of falls and wandering behaviors without compromising user privacy. In addition, AI-powered analytics enhance the accuracy of detection by processing microradar data to identify anomalies in movement patterns. To support real-time caregiver intervention, our system uses Web Real-Time Communication (WebRTC) and peer-to-peer (P2P) communication for seamless alerts. By addressing these limitations and leveraging interdisciplinary advancements, our framework offers a comprehensive, privacy-conscious, and scalable solution for the monitoring of patients with ADRD. Different aspects pertaining to RQ 4 are discussed in the following subsections.

#### Ensuring Patient Safety

Patient safety is the cornerstone of any remote monitoring system for patients with AD. Wearable devices such as smartwatches and fitness trackers can monitor crucial health indicators such as gait, sleep patterns, physical activity, and falls. Environmental sensors placed in the patient’s home can track temperature, motion, and other conditions to detect unusual behavior, such as wandering or falls, ensuring that immediate action can be taken [72].

Given the sensitivity of health data, robust security measures are essential. Data from wearable devices and sensors must be encrypted to prevent unauthorized access. Compliance with regulations such as the GDPR and HIPAA is necessary to protect patient privacy. Strong authentication methods and regular software updates can help maintain data integrity and security.

#### Ease of Use for Caregivers

The system must be user-friendly, as caregivers for individuals with ADRD may not be technologically savvy. A simple, intuitive interface is crucial, allowing for straightforward navigation and clear, actionable alerts to reduce ambiguity and stress. Customizable notifications and automated alerts ensure that caregivers are informed of critical issues without needing to constantly monitor the system while minimizing false alarms to avoid unnecessary stress. In addition, providing educational resources within the system helps caregivers understand how to best use the monitoring tools and improve their caregiving skills. This approach empowers caregivers, enhances efficiency, and improves patient safety by facilitating timely interventions [73,74].

#### Ethical Considerations

Ethical considerations for a potential remote monitoring framework for patients with AD include ensuring informed consent and respecting patient autonomy. Considering cognitive impairments, obtaining informed consent must involve patients as much as possible as well as legal representatives or caregivers when necessary to ensure understanding and voluntary participation. Privacy and confidentiality are paramount, requiring compliance with data protection regulations and secure data handling practices. The system should be minimally intrusive, using discreet technologies to balance safety with patient dignity and avoiding constant visible monitoring that could lead to a loss of independence. Transparency in communication about the system’s operation and data use, as well as providing feedback mechanisms, builds trust and addresses concerns. Ethical design principles involving user-centered approaches and bias mitigation in AI, alongside regular ethical reviews and oversight by ethics committees, ensure that the system remains respectful and patient centered while enhancing safety [75,76].

#### Compatibility With Existing Systems

To be effective, the remote monitoring system for patients with AD must integrate seamlessly with existing health care infrastructure, such as electronic health records (EHRs) [77]. This integration ensures that all relevant health data from the monitoring system are accurately and efficiently captured within the broader health care framework. Using standardized protocols and application programming interfaces is critical for facilitating data sharing and interoperability [78]. These standards, such as Health Level Seven Fast Healthcare Interoperability Resources, ensure that data from the monitoring system can be easily and securely exchanged with different EHR systems and other health IT platforms [79]. Seamless integration allows physicians, specialists, and other clinicians to access comprehensive, up-to-date information about patients, promoting coordinated care. For instance, if a remote monitoring system detects a fall or wandering incident, this information can be automatically logged in the patient’s EHR, alerting HCPs in real time. This ensures that primary care physicians, specialists, and hospital staff have a unified view of the patient’s health status and recent events, enabling more informed decision-making and timely interventions.

Moreover, compatibility with existing systems helps in reducing duplication of efforts and minimizes the risk of errors. HCPs can avoid manually entering data, which not only saves time but also reduces the likelihood of transcription errors. By ensuring that the remote monitoring data are automatically integrated into EHRs, the system enhances the accuracy and reliability of patient records. In addition to technical interoperability, organizational processes and workflows must be considered to ensure smooth integration. Training for physicians, nurses, and other HCPs on how to use the new system and interpret its data within the context of existing EHRs is essential. Developing clear protocols for how and when data from the monitoring system should be used in patient care can help ensure that the system is used effectively. By enabling seamless data sharing and integration, the system supports a more coordinated and comprehensive approach to health care, ultimately improving outcomes for patients with AD and reducing the burden on caregivers and medical professionals.

#### Scalability and Flexibility

A scalable system is essential for accommodating the increasing number of patients with AD who require care. As demand for remote monitoring solutions grows, the system must expand without compromising performance or reliability. This need involves designing the system architecture to support a larger user base and additional data processing and storage capabilities. Scalable infrastructure ensures that the system can handle a surge in the number of patients and caregivers, allowing health care providers to maintain high standards of care even as the user population increases. Moreover, incorporating standardized interfaces and protocols such as application programming interfaces and interoperability standards ensures that the system can easily connect with new devices and technologies, facilitating continuous improvement and innovation [80].

Flexibility in the remote monitoring system is equally important. The system should have a modular architecture that allows for the easy integration of new sensors and technologies as they become available. This modular approach ensures that the system can adapt to technological advancements and incorporate innovative solutions without requiring a complete redesign. It should also be configurable to meet the specific requirements of various health care settings, such as in-home care, assisted living facilities, or hospitals, providing tailored solutions that address the unique challenges of each environment. The system’s scalability and flexibility ensure that it adapts to evolving health care needs, enhancing patient care and supporting the growth of remote monitoring technologies [80].

#### Technology Components

Wearable technology is essential for health monitoring, allowing for the real-time tracking of multiple health variables such as heart rate, physical activity, sleep habits, and location [56,72]. These devices help monitor the patient’s daily routines and identify emergencies such as falls. Environmental sensors also enhance the patient’s living environment by monitoring door activity, motion, and room temperature to detect unusual activity. For instance, if a patient leaves the house at an unusual time, an alert can be issued to the caregiver. Smartphone apps enable caregivers to easily monitor and receive notifications, enter observations, create personalized alerts, and access learning materials. Continuous connectivity ensures that caregivers are always informed about the patient’s status.

Implementing AI and machine learning algorithms to analyze data collected from wearables and other devices allows for the identification of trends indicating potential health issues. These predictive analytics enable caregivers to anticipate problems and take preventive measures before they become critical, thus improving patient care and safety. Moreover, an IoT infrastructure is essential for ensuring seamless data transfer between all connected devices, with cloud computing offering scalable processing and storage capacity and edge computing minimizing delays by processing data locally. Effective data management is crucial for making sense of the vast amounts of data generated by the system, allowing for trend analysis and informed clinical decisions. Advanced analytics techniques can also provide personalized patient care. In addition, robust security technologies such as intrusion detection systems, firewalls, and encryption methods are vital to protect the system from threats, with frequent maintenance and updates ensuring ongoing security

#### Comprehensive Framework

##### Overview

**Figure 4:**
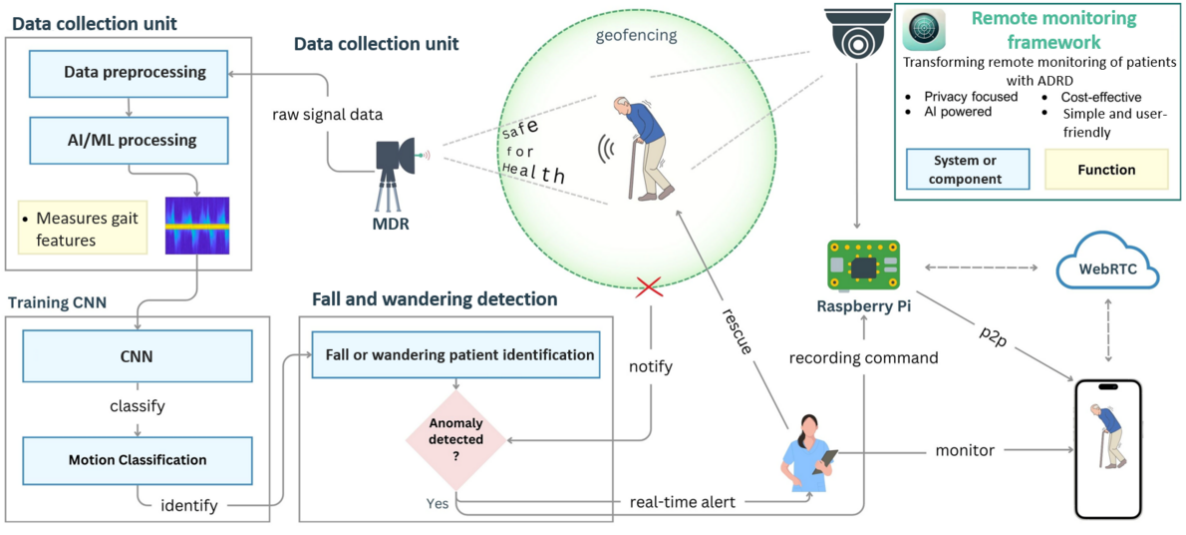
Schematic framework for remote monitoring of patients with Alzheimer disease. ADRD: Alzheimer disease and related dementias; AI: artificial intelligence; CNN: convolutional neural network; MDR: micro-Doppler radar; ML: machine learning; P2P: peer to peer; WebRTC: Web Real-Time Communication.

On the basis of the discussions in the previous subsections and the findings of previous studies, we propose a comprehensive remote monitoring framework, as shown in Figure 4, by incorporating the use of wearable technologies, sensors, AI-powered analytics, and a safe IoT infrastructure. The elements in the schematic framework are intricately connected and together create a comprehensive system for the remote monitoring of patients with AD. The framework is designed with a strong focus on health and safety. The use of microradar technology ensures that monitoring is nonintrusive and safe for patients, reducing the stress associated with traditional monitoring systems. This framework not only enhances patient safety but also alleviates the burden on caregivers by providing reliable, real-time support and intervention tools. The following is a detailed discussion of how each point contributes to the overall monitoring framework.

##### Data Collection

The data collection unit is the central component of the monitoring system and is responsible for gathering and processing the data required to track the patient’s movements and health status effectively. At its core, the unit uses micro-Doppler radar technology to capture raw signal data by continuously monitoring the patient’s movements in a nonintrusive manner [81]. This technology is crucial as it does not require the patient to wear any devices or interact with the system, thereby preserving the patient’s privacy and minimizing any stress or discomfort that might arise from traditional monitoring systems. The raw data collected by the micro-Doppler radar undergoes a critical preprocessing stage in which essential gait features are extracted. This step is vital in filtering out noise and isolating the most relevant aspects of the patient’s movements that could signal potential risks. Once preprocessed, the data are fed into advanced AI and machine learning algorithms, which analyze the extracted features to identify patterns in the patient’s movements. This sophisticated processing enables the system to distinguish between normal daily activities and potentially hazardous situations such as falls or wandering, thereby enhancing the system’s ability to ensure the patient’s safety while maintaining their dignity and comfort.

##### Data Processing

The data processing component plays a critical role in enhancing the system’s ability to accurately classify various patient movements, which is fundamental to ensuring effective monitoring and maintaining the safety of individuals with ADRD. The process begins with preprocessed data being fed into a convolutional neural network [82], a powerful AI model specifically designed to recognize and analyze patterns within the input data. This network is trained to classify different types of motions, enabling it to distinguish between normal daily activities such as walking or sitting and potentially hazardous incidents that may require immediate intervention, such as falls or wandering outside a designated safe zone. The convolutional neural network’s ability to make these distinctions is vital to the system’s overall accuracy and reliability as it allows for the early detection of unusual or dangerous behaviors. This, in turn, facilitates timely alerts and responses, ensuring that caregivers can intervene quickly and appropriately to protect the patient’s well-being. Through this advanced data processing approach, the system not only enhances patient safety but also provides peace of mind to caregivers through the knowledge that the system is capable of accurately monitoring and responding to the specific needs of patients with ADRD.

##### Fall and Wandering Detection

The fall and wandering detection component is a critical part of the monitoring system, designed to ensure the continuous safety of patients by closely tracking their movements and identifying any signs of falling or wandering outside a predefined safe area. The system uses a geofencing approach in which a *defined* boundary or safe zone is established around the patient’s environment. This safe zone is carefully configured based on the patient’s needs and typical movement patterns. The system continuously monitors the patient’s movements within this boundary using sophisticated pattern identification techniques. By analyzing these movement patterns in real time, the system can quickly detect any deviations from the norm. For example, if the patient crosses this virtual boundary or exhibits movements that suggest a fall, such as a sudden loss of balance or an abrupt change in posture, the system immediately flags this behavior as an anomaly. This continuous, vigilant monitoring is essential for identifying potential risks before they can lead to serious harm.

Once an anomaly is detected, the system’s response should be both swift and automated. The anomaly detection process should be finely tuned to recognize specific triggers, such as the patient moving outside the safe zone or experiencing a fall. When such an event is identified, the system automatically triggers an alert, ensuring that caregivers are notified in real time. These alerts should be designed to reach caregivers through various channels, such as mobile notifications or direct messages, providing them with immediate information about the patient’s situation. This real-time notification system is crucial for enabling caregivers to respond promptly and appropriately to any incidents, whether it involves assisting the patient after a fall or guiding them back to safety after wandering. The combination of continuous monitoring and instantaneous alerting forms a robust safety net, significantly reducing the risks associated with falls and wandering in patients with ADRD.

##### Caregiver Notification and Support

The caregiver notification and support component is designed to empower caregivers with the tools and information they need to effectively monitor and respond to the needs of patients in real time. Central to this component are real-time alerts, which are instantly sent to caregivers’ devices, ensuring that they are always aware of the patient’s status. These immediate notifications are particularly crucial in emergency situations, such as when a fall or wandering event occurs. By receiving alerts the moment an anomaly is detected, caregivers can intervene promptly, providing necessary assistance or preventing further harm to the patient. This real-time communication is key to maintaining the safety and well-being of patients with ADRD.

In addition, the system is equipped with a recording command and rescue feature facilitated by a Raspberry Pi module. This module plays a vital role by capturing and recording the event as soon as an anomaly is detected. The recorded data are automatically sent to caregivers, offering them detailed insights into the incident. This documentation not only aids in immediate rescue actions but also serves as a valuable resource for later analysis. By reviewing recorded incidents, caregivers and medical professionals can better understand the circumstances leading up to the event, which is essential for medical evaluations and refining future care strategies. The ability to document and analyze these events ensures that care can be continuously improved, ultimately enhancing the quality of life of both patients and caregivers.

##### WebRTC and P2P Communication

The framework integrates advanced communication technologies to ensure seamless monitoring and interaction between caregivers and the system, providing a reliable and immediate connection regardless of location. A key component of this system is the use of WebRTC technology, which facilitates real-time P2P communication [83,84]. This technology allows caregivers to monitor the patient from their smartphones or other devices with ease and without delay, ensuring that they remain connected to the patient’s status at all times. The use of WebRTC ensures that the communication is not only immediate but also direct, reducing latency and enhancing the responsiveness of the system.

This capability significantly enhances patient safety as it allows caregivers to maintain a continuous, real-time connection with the system, enabling them to respond quickly to any situation that may arise. Whether it is monitoring routine activities or responding to alerts triggered by the system, WebRTC ensures that caregivers can provide timely and effective interventions. The seamless monitoring facilitated by this technology is crucial for reinforcing the safety net around patients with ADRD, giving caregivers the confidence and tools they need to manage their loved ones’ care effectively, no matter where they are.

### Conclusions

This study highlights the revolutionary potential of remote monitoring technologies in AD care, emphasizing the critical balance among innovation, practicality, and empathy. The comprehensive analysis revealed that, while wearable devices and ambient sensors significantly enhance the ability to track and support patients with AD, they also introduce challenges related to privacy, data security, and user acceptance. The findings stress the necessity of involving patients and caregivers in the design process to ensure that these technologies meet their specific needs and regulatory requirements, which is crucial to the successful implementation of these technologies.

The proposed remote monitoring framework integrates cutting-edge microradar technology with AI-powered processing to create a nonintrusive, privacy-conscious solution for monitoring patients with AD and dementia. By focusing on real-time data processing, caregiver notification, and seamless communication, the system not only enhances patient safety but also provides critical support to caregivers, ultimately improving the quality of care for individuals with ADRD. Future research can focus on refining these systems to enhance their usability, accessibility, and security, thereby improving the overall caregiving experience and patient well-being. By advancing remote monitoring practices, we can create a supportive environment that alleviates caregiver burdens and promotes a higher quality of life for individuals living with AD.

## Appendix

Multimedia Appendix 1 PRISMA (Preferred Reporting Items for Systematic Reviews and Meta-Analyses) checklist.

Multimedia Appendix 2 Remote monitoring technologies using wearable and portable device–based sensors.

Multimedia Appendix 3 Remote monitoring technologies using nonwearable home sensors.

Multimedia Appendix 4 Remote monitoring technologies for dementia care and their role in cognitive assessment.

Multimedia Appendix 5 Different privacy and security considerations in remote monitoring.

## Abbreviations

AD: Alzheimer disease
ADRD: Alzheimer disease and related dementias
AI: artificial intelligence
BPSD: behavioral and psychological symptoms of dementia
EHR: electronic health record
GDPR: General Data Protection Regulation
HCP: health care professional
HIPAA: Health Insurance Portability and Accountability Act
IoMT: Internet of Medical Things
IoT: Internet of Things
MCI: mild cognitive impairment
mHealth: mobile health
P2P: peer to peer
PRISMA: Preferred Reporting Items for Systematic Reviews and Meta-Analyses
RQ: research question
WebRTC: Web Real-Time Communication
