# Supplementary material for: Advancing Remote Monitoring for Patients With Alzheimer Disease and Related Dementias: Systematic Review
Source: JMIR Aging. 2025 May 14;8:e69175. doi: 10.2196/69175 (PMC12120371; doi:10.2196/69175)
Supplement: Multimedia Appendix 2 [file aging_v8i1e69175_app2.docx]

**Table S1.** Remote Monitoring techniques using wearable and portable device-based sensors

| Type of Data | Data Collection Process | Application | Ref. |
| --- | --- | --- | --- |
| Gait parameters (speed, variability, and stride length) | Devices were on the waist or ankle embedded with accelerometers and gyroscopes | Data helps in maintaining the levels of physical activity and energy utilization | [32] |
| Physical activity | Accelerometer embedded in wearables captures the data of physical activities including steps taken, intensity of activity, and duration of activity. | Tracking and maintaining physical activities shows slow progression of AD. Helps in the continuous monitoring of blood pressure | [32,38] |
| Physiological data (daily activity and sleep pattern) | Daily activity: wristbands, smart clothes, smartphones, smartwatches, armbands, or chest straps.  Sleep: Basis Health Tracker, the Misfit Shine, the Fitbit Flex, the Withings PulseO2, and the Actiwatch Spectrum actigraph. | Continuous monitoring of vulnerable patients | [39] |
| Sleep | A dream headband is used during sleep that records, stores, and automatically analyze physiological data in real time. | Helps in monitoring daily sleep patterns and physiological data at home and without constraint able to freely access their reports. | [40] |
| Social behavior | Smartphone devices to assess social behavior, via monitoring calls, text messages, or internet browsing. | Helps to determine the social activity and level and keep them from isolation | [42] |
| Temperature, Blood pressure, Heart Rate, Glucose Levels | Novel sensors are developed and use wireless methods like Bluetooth, Wi-Fi, NFC, and ZigBee to access the captured data from the cloud. | Helps in the management of devices and real-time data monitoring and storing the data securely. | [43] |
| Prescription planning data | IoT-based tool that helps in medication intake. | Intervention by reminding to take medication according to the prescription plan. | [44] |
| Physiological data | A proposed semantic framework is used to collect the data with multiple sensors | Help in determining the disease progression and timely alerts with real-time data monitoring. | [45] |
| Physiological data | From the monitoring devices data is collected and processed into content prediction, behavior recognition, and content recognition | It is a computational model that covers the proposed objectives for general monitoring, data processing, and sending alerts to the caregiver in case of danger to the patient | [46] |
| Social life, psychological health | Devices such as headsets, smart glasses, and new generations of smartphones. | Enable more immersive experiences in various aspects of everyday life, including web-based home shopping, leisure activities, and communication. | [47] |
| Inage and video based data | Machine learning algorithms are used for recognition and detection using training and test data. | It helps in assisting patients with dementia and memory loss. | [48] |
| Location based data | It uses GPS and Bluetooth to track the location and also give medication alerts. | It helps caregivers to track the patient and check their timely medication. | [48] |
| Blood volume pressure, Interbeat interval , Heart Rate, Electrodermal activity, Skin temperature | Data was collected from the multiple sensors implanted in the wristband and with the assistance of caregivers. | To collect physiological and behavioral data from elderly people affected by Alzheimer’s during their everyday life, and to explore opportunities to find relationships among the physiological trends and the onset of crises. | [49] |
| Cognitive abilities and other data | Data collected from wrist-worn actigraphy device ActiGraph wGT3X | Machine algorithms were used for training and testing the data acquired to predict the risks and progression of the disease | [50] |

**References:**

[32] Popp Z, Low S, Igwe A, Rahman MS, Kim M, Khan R, et al. Shifting From Active to Passive Monitoring of Alzheimer Disease: The State of the Research. J Am Heart Assoc 2024;13. https://doi.org/10.1161/JAHA.123.031247/ASSET/24EDDB97-F7FF-408E-B73F-DFE258A7B0E2/ASSETS/GRAPHIC/JAH39047-FIG-0002.PNG.

[38] Fares N, Sherratt RS, Elhajj IH. Directing and Orienting ICT Healthcare Solutions to Address the Needs of the Aging Population. Healthcare 2021, Vol 9, Page 147 2021;9:147. https://doi.org/10.3390/HEALTHCARE9020147.

[39] Anghel I, Cioara T, Moldovan D, Antal M, Pop CD, Salomie I, et al. Smart Environments and Social Robots for Age-Friendly Integrated Care Services. International Journal of Environmental Research and Public Health 2020, Vol 17, Page 3801 2020;17:3801. https://doi.org/10.3390/IJERPH17113801.

[40] Chouraki A, Tournant J, Arnal P, Pépin JL, Bailly S. Objective multi-night sleep monitoring at home: variability of sleep parameters between nights and implications for the reliability of sleep assessment in clinical trials. Sleep 2023;46. https://doi.org/10.1093/SLEEP/ZSAC319.

[42] Read E, Woolsey C, Donelle L, Weeks L, Chinho N. Passive Remote Monitoring and Aging in Place: A Scoping Review. Can J Aging 2023;42:20–32. https://doi.org/10.1017/S0714980822000198.

[43] Shiwani T, Relton S, Evans R, Kale A, Heaven A, Clegg A, et al. New Horizons in artificial intelligence in the healthcare of older people. Age Ageing 2023;52. https://doi.org/10.1093/AGEING/AFAD219.

[44] Purohit P, Khanpara P, Patel U, Kathiria P. IoT based Ambient Assisted Living Technologies for Healthcare: Concepts and Design Challenges. 6th International Conference on I-SMAC (IoT in Social, Mobile, Analytics and Cloud), I-SMAC 2022 - Proceedings 2022:111–6. https://doi.org/10.1109/I-SMAC55078.2022.9987375.

[45] Giannios G, Mpaltadoros L, Alepopoulos V, Grammatikopoulou M, Stavropoulos TG, Nikolopoulos S, et al. A Semantic Framework to Detect Problems in Activities of Daily Living Monitored through Smart Home Sensors. Sensors 2024, Vol 24, Page 1107 2024;24:1107. https://doi.org/10.3390/S24041107.

[46] Machado SD, da Rosa Tavares JE, Martins MG, Barbosa JLV, González GV, Leithardt VRQ. Ambient Intelligence Based on IoT for Assisting People with Alzheimer’s Disease Through Context Histories. Electronics 2021, Vol 10, Page 1260 2021;10:1260. https://doi.org/10.3390/ELECTRONICS10111260.

[47] Abdi S, de Witte L, Hawley M. Emerging Technologies With Potential Care and Support Applications for Older People: Review of Gray Literature. JMIR Aging 2020;3:e17286. https://doi.org/10.2196/17286.

[48] Patil S, Shobha T, Janet Kumari JJ, Khanum R, Anjum R, Manjunath S, et al. Medical Assistance for Alzheimer’s Disease Using Smart Specs. 4th International Conference on Circuits, Control, Communication and Computing, I4C 2022 2022:363–6. https://doi.org/10.1109/I4C57141.2022.10057684.

[49] Amato F, Crovari P, Masciadri A, Bianchi S, Pasquarelli MGG, Toldo M, et al. Clone: A promising system for the remote monitoring of Alzheimer’s patients an experimentation with a wearable device in a village for Alzheimer’s care. ACM International Conference Proceeding Series 2018:255–60. https://doi.org/10.1145/3284869.3284906.

[50] Cho E, Kim S, Heo SJ, Shin J, Hwang S, Kwon E, et al. Machine learning-based predictive models for the occurrence of behavioral and psychological symptoms of dementia: model development and validation. Scientific Reports 2023 13:1 2023;13:1–12. https://doi.org/10.1038/s41598-023-35194-5.
