# Supplementary material for: Advancing Remote Monitoring for Patients With Alzheimer Disease and Related Dementias: Systematic Review
Source: JMIR Aging. 2025 May 14;8:e69175. doi: 10.2196/69175 (PMC12120371; doi:10.2196/69175)
Supplement: Multimedia Appendix 3 [file aging_v8i1e69175_app3.docx]

**Table S2**. Remote Monitoring techniques using non-wearable home sensors

| Type of Data | Data Collection Process | Application | Ref. |
| --- | --- | --- | --- |
| Gait | Infrared sensors placed in the home detects the individual moments while walking and provide insights on gait characteristics. | Assessment of characteristics like walking speed, rhythm, and variability. | [32] |
| Sleep | Wireless mattress sensors and home-based ambient sensors are used to track sleep time, movements, and sleep patterns. | Helps in detecting mild cognitive impairment and early stages of AD. | [32] |
|  | A night monitoring system with motion detectors is used to monitor sleep patterns. | In unusual behavior, the detectors will alert the caregivers for assistance. | [42] |
| Physical activity | Collection of indoor movement using infrared sensors. | It allows continuous monitoring of active levels without the participation of the individual. | [32,41] |
|  | Sound sensors, video cameras, RFID readers, pressure sensors, luminosity sensors, proximity sensors, pressure sensors, temperature sensors, humidity sensors, and location tracking sensors used to capture activities | The data helps in the assessment of disease progression. | [38] |
| Behavior patterns | Sensor installed in kitchen, bedroom, bathrooms, fall detection sensors, etc. | helps in tracking mobility stability, frequency, and time spent in different areas of the home. | [32] |
| Fall detection | Microsoft Kinect camera is used for gait monitoring to detect falls among the elderly | the video data helps in the assessment of falls and reduces the falls among elder people. | [42] |
|  | Motion sensor-activated light path that was activated when the participant would get out of bed | Helps in reducing the falls during nighttime. |  |
| Bed sensor | Data collected from Bed Pressure Sensors | Helps in sleep problem detection. | [39] |
| Driving pattern | Using a global positioning system preclinical AD data is collected and compared with the progression of AD. | Help in capturing the difference in behavior changes associated with AD. | [32] |
| People activities | Data is collected from different devices embedded with sensors. | Uses data mining and predictive models for predicting problems to make health care decisions. | [51] |
| Sleep pattern | Data is collected from different devices embedded with sensors. | Provide clinicians with early warnings of health deteriorations or relapses, enabling timely interventions and improved patient outcomes. | [52] |
| Speech pattern | Analyzes the data captured from health records via calls and tests. | Provide a diagnosis for Parkinson’s and Alzheimer’s disease. | [43] |
| X-ray and CT scan, muscular degeneration | AI scanning images and video processing. | Help in diagnosing cancer from scans and degeneration from fundus photography. | [50] |
| Social engagement | Solidarity feeling of the elderly through conversation, quizzes, tongue twisters, and arithmetic calculations. | This result shows promise in social engagement and provides a companion that eliminates isolation among elders. | [51] |
| Medical prompt and emotional support | AI-powered robots by accessing the environment and user-specific characteristics. | Helps in physical rehabilitation and deisolation. | [43,47] |
| Intervention of robots | Robots named Nao, Pepper, and PARO for cognitively impaired patients with dementia. | Helps in the detection of behavioral disturbances, physical exercises tutoring, recreational activities, stress management, companion for older adults, rehabilitation recreational activities; sentiment analysis, narrative-memory-based human-robot companion, medicine taking reminding. | [39] |

**References:**

[32] Popp Z, Low S, Igwe A, Rahman MS, Kim M, Khan R, et al. Shifting From Active to Passive Monitoring of Alzheimer Disease: The State of the Research. J Am Heart Assoc 2024;13. https://doi.org/10.1161/JAHA.123.031247/ASSET/24EDDB97-F7FF-408E-B73F-DFE258A7B0E2/ASSETS/GRAPHIC/JAH39047-FIG-0002.PNG.

[38] Fares N, Sherratt RS, Elhajj IH. Directing and Orienting ICT Healthcare Solutions to Address the Needs of the Aging Population. Healthcare 2021, Vol 9, Page 147 2021;9:147. https://doi.org/10.3390/HEALTHCARE9020147.

[39] Anghel I, Cioara T, Moldovan D, Antal M, Pop CD, Salomie I, et al. Smart Environments and Social Robots for Age-Friendly Integrated Care Services. International Journal of Environmental Research and Public Health 2020, Vol 17, Page 3801 2020;17:3801. https://doi.org/10.3390/IJERPH17113801.

[41] Tong K, Attenborough K, Sharp D, Taherzadeh S, Deepak-Gopinath M, Vseteckova J. Acceptability of Remote Monitoring in Assisted Living/Smart Homes in the United Kingdom and Associated Use of Sounds and Vibrations—A Systematic Review. Applied Sciences (Switzerland) 2024;14:843. https://doi.org/10.3390/APP14020843/S1.

[42] Read E, Woolsey C, Donelle L, Weeks L, Chinho N. Passive Remote Monitoring and Aging in Place: A Scoping Review. Can J Aging 2023;42:20–32. https://doi.org/10.1017/S0714980822000198.

[43] Shiwani T, Relton S, Evans R, Kale A, Heaven A, Clegg A, et al. New Horizons in artificial intelligence in the healthcare of older people. Age Ageing 2023;52. https://doi.org/10.1093/AGEING/AFAD219.

[44] Purohit P, Khanpara P, Patel U, Kathiria P. IoT based Ambient Assisted Living Technologies for Healthcare: Concepts and Design Challenges. 6th International Conference on I-SMAC (IoT in Social, Mobile, Analytics and Cloud), I-SMAC 2022 - Proceedings 2022:111–6. https://doi.org/10.1109/I-SMAC55078.2022.9987375.

[47] Abdi S, de Witte L, Hawley M. Emerging Technologies With Potential Care and Support Applications for Older People: Review of Gray Literature. JMIR Aging 2020;3:e17286. https://doi.org/10.2196/17286.

[50] Cho E, Kim S, Heo SJ, Shin J, Hwang S, Kwon E, et al. Machine learning-based predictive models for the occurrence of behavioral and psychological symptoms of dementia: model development and validation. Scientific Reports 2023 13:1 2023;13:1–12. https://doi.org/10.1038/s41598-023-35194-5.

[51] Sapci AH, Sapci HA. Innovative Assisted Living Tools, Remote Monitoring Technologies, Artificial Intelligence-Driven Solutions, and Robotic Systems for Aging Societies: Systematic Review. JMIR Aging 2019;2:e15429. https://doi.org/10.2196/15429.

[52] Li C, Wang J, Wang‎ S, Zhang Y. A review of IoT applications in healthcare. Neurocomputing 2024;565:127017. https://doi.org/10.1016/J.NEUCOM.2023.127017.
