# Supplementary material for: Advancing Remote Monitoring for Patients With Alzheimer Disease and Related Dementias: Systematic Review
Source: JMIR Aging. 2025 May 14;8:e69175. doi: 10.2196/69175 (PMC12120371; doi:10.2196/69175)
Supplement: Multimedia Appendix 4 [file aging_v8i1e69175_app4.docx]

**Table S3:** Remote Monitoring Technologies for Dementia Care and Their Role in Cognitive Assessment

| Technology Type | Primary Goals | Effectiveness | Relevance to Clinicians | Relevance to Caregivers | Cognitive Monitoring Applications | Reference |
| --- | --- | --- | --- | --- | --- | --- |
| Wearable Sensors | Continuous monitoring of vital signs, activity, and sleep patterns | Improves early detection of health deterioration and adherence to treatment plans | Helps in diagnosing early-stage AD/ADRD symptoms and tracking disease progression | Provides real-time alerts for abnormal vitals or movement, reducing supervision burden | Emerging wearable EEG devices can assess cognitive changes and neurofeedback in real-time | [53,54] |
| Home-based Sensors | Detects movement patterns, fall risks, and sleep disturbances | Effective in tracking daily activity trends and recognizing abnormal behaviors | Supports clinical decision-making with objective behavioral data | Helps detect falls or wandering, allowing timely intervention | Passive monitoring of activity levels may correlate with cognitive decline | [41,50,55] |
| AI & Machine Learning | Predicts cognitive decline, analyzes speech and movement data | Machine learning models show high accuracy in symptom progression prediction | Aids in early diagnosis and personalized intervention strategies | Provides caregivers with predictive alerts for behavior changes | AI models analyze speech, typing, and gaze patterns to assess cognitive function and drug response | [32,38,50,51] |
| Robotics (Assistive & Social) | Supports cognitive engagement, emotional well-being, and daily tasks | Reduces patient agitation, enhances interaction, and improves mental stimulation | Acts as a supplementary tool for patient interaction and rehabilitation | Alleviates stress by providing companionship and basic assistance | Robots can facilitate cognitive exercises, memory recall activities, and conversational assessments | [42,43,47,51] |
| mHealth Apps | Medication reminders, cognitive exercises, and emergency alerts | Increases patient adherence to prescribed care routines | Supports remote monitoring and improves medication compliance | Reduces the burden of daily task management for caregivers | Apps can include digital cognitive assessments and therapy tracking for treatment efficacy | [56] |
| Internet of Medical Things (IoMT) | Secure and integrated patient monitoring via connected devices | Enhances real-time data accessibility and interoperability with EHRs | Provides seamless health tracking and patient record integration | Ensures automated alerts and remote supervision capabilities | Enables real-time cognitive data collection for clinical trials and personalized interventions | [57,58] |

References:

[32] Popp Z, Low S, Igwe A, Rahman MS, Kim M, Khan R, et al. Shifting From Active to Passive Monitoring of Alzheimer Disease: The State of the Research. J Am Heart Assoc 2024;13. https://doi.org/10.1161/JAHA.123.031247/ASSET/24EDDB97-F7FF-408E-B73F-DFE258A7B0E2/ASSETS/GRAPHIC/JAH39047-FIG-0002.PNG.

[38] Fares N, Sherratt RS, Elhajj IH. Directing and Orienting ICT Healthcare Solutions to Address the Needs of the Aging Population. Healthcare 2021, Vol 9, Page 147 2021;9:147. https://doi.org/10.3390/HEALTHCARE9020147.

[41] Tong K, Attenborough K, Sharp D, Taherzadeh S, Deepak-Gopinath M, Vseteckova J. Acceptability of Remote Monitoring in Assisted Living/Smart Homes in the United Kingdom and Associated Use of Sounds and Vibrations—A Systematic Review. Applied Sciences (Switzerland) 2024;14:843. https://doi.org/10.3390/APP14020843/S1.

[42] Read E, Woolsey C, Donelle L, Weeks L, Chinho N. Passive Remote Monitoring and Aging in Place: A Scoping Review. Can J Aging 2023;42:20–32. https://doi.org/10.1017/S0714980822000198.

[43] Shiwani T, Relton S, Evans R, Kale A, Heaven A, Clegg A, et al. New Horizons in artificial intelligence in the healthcare of older people. Age Ageing 2023;52. https://doi.org/10.1093/AGEING/AFAD219.

[47] Abdi S, de Witte L, Hawley M. Emerging Technologies With Potential Care and Support Applications for Older People: Review of Gray Literature. JMIR Aging 2020;3:e17286. https://doi.org/10.2196/17286.

[50] Cho E, Kim S, Heo SJ, Shin J, Hwang S, Kwon E, et al. Machine learning-based predictive models for the occurrence of behavioral and psychological symptoms of dementia: model development and validation. Scientific Reports 2023 13:1 2023;13:1–12. https://doi.org/10.1038/s41598-023-35194-5.

[51] Sapci AH, Sapci HA. Innovative Assisted Living Tools, Remote Monitoring Technologies, Artificial Intelligence-Driven Solutions, and Robotic Systems for Aging Societies: Systematic Review. JMIR Aging 2019;2:e15429. https://doi.org/10.2196/15429.

[53] Godkin FE, Turner E, Demnati Y, Vert A, Roberts A, Swartz RH, et al. Feasibility of a continuous, multi-sensor remote health monitoring approach in persons living with neurodegenerative disease. J Neurol 2022;269:2673–86. https://doi.org/10.1007/S00415-021-10831-Z/TABLES/2.

[54] Stavropoulos TG, Lazarou I, Diaz A, Gove D, Georges J, Manyakov N V., et al. Wearable Devices for Assessing Function in Alzheimer’s Disease: A European Public Involvement Activity About the Features and Preferences of Patients and Caregivers. Front Aging Neurosci 2021;13:643135. https://doi.org/10.3389/FNAGI.2021.643135/BIBTEX.

[55] Tian YJ, Felber NA, Pageau F, Schwab DR, Wangmo T. Benefits and barriers associated with the use of smart home health technologies in the care of older persons: a systematic review. BMC Geriatrics 2024 24:1 2024;24:1–16. https://doi.org/10.1186/S12877-024-04702-1.

[56] Lazarou I, Stavropoulos TG, Mpaltadoros L, Nikolopoulos S, Koumanakos G, Tsolaki M, et al. Human Factors and Requirements of People with Cognitive Impairment, Their Caregivers, and Healthcare Professionals for mHealth Apps Including Reminders, Games, and Geolocation Tracking: A Survey-Questionnaire Study. J Alzheimers Dis Rep 2021;5:497–513. https://doi.org/10.3233/ADR-201001.

[57] Pou-Prom C, Raimondo S, Rudzicz F. A Conversational Robot for Older Adults with Alzheimer’s Disease. ACM Transactions on Human-Robot Interaction (THRI) 2020;9. https://doi.org/10.1145/3380785.

[58] Mathkor DM, Mathkor N, Bassfar Z, Bantun F, Slama P, Ahmad F, et al. Multirole of the internet of medical things (IoMT) in biomedical systems for managing smart healthcare systems: An overview of current and future innovative trends. J Infect Public Health 2024;17:559–72. https://doi.org/10.1016/J.JIPH.2024.01.013.
