# Supplementary material for: Advancing Remote Monitoring for Patients With Alzheimer Disease and Related Dementias: Systematic Review
Source: JMIR Aging. 2025 May 14;8:e69175. doi: 10.2196/69175 (PMC12120371; doi:10.2196/69175)
Supplement: Multimedia Appendix 5 [file aging_v8i1e69175_app5.docx]

**Table S4:** Different privacy and security considerations in remote monitoring

| Domain | Focus Area | Purpose | Examples |
| --- | --- | --- | --- |
| Ethical and Legal | Ethical Considerations | Ensures patient rights, safety, and ethical handling of data. | Patient Safety, Privacy |
|  | Privacy Regulations | Provides legal frameworks for data protection and compliance. | GDPR, HIPAA |
| Technical Safeguards | Security Layers | Implements protective measures to secure data integrity and confidentiality. | Encryption, Access Control, Audits, Secure Protocols |
|  | Potential Threats & Countermeasures | Identifies risks and strategies to prevent or mitigate security breaches. | End-to-End Encryption, Rapid Incident Response |
| Data Handling | Data Sensitivity | Recognizes the importance of protecting different types of sensitive data. | Personal Data, Medical Records, Behavioral Patterns |
|  | AI and Machine Learning | Focuses on anonymizing and securely processing data for analysis while ensuring privacy. | Anonymized Data |
| System Vulnerabilities | IoT Vulnerabilities | Highlights security risks in interconnected devices used for data collection. | Smartphones, Wearables, Sensors |
